# *“I feel myself incomplete, and I am inferior to people”:* experiences of Sudanese women living with obstetric fistula in Khartoum, Sudan

**DOI:** 10.1186/s12978-019-0846-y

**Published:** 2019-12-21

**Authors:** Salma A. E. Ahmed, Viva C. Thorsen, Salma A. E. Ahmed

**Affiliations:** Institute of Health and Society, Department of Community Medicine and Global Health, University of Oslo, P.O. Box 1130 Blindern, 0318 Oslo, Norway

**Keywords:** Obstetric fistula, Experiences, Maternal morbidity, Stigma, Coping mechanisms, Sudan

## Abstract

**Background:**

Obstetric fistula is among the most devastating maternal morbidities that occur as a result of prolonged, obstructed labor. Usually, the child dies in a large number of the cases. Moreover, some of the women become infertile while the majority suffer physical, psychosocial and economic challenges. Approximately 5000 new cases of obstetric fistula occur in Sudan each year. However, their experiences are under documented. Therefore, this study aimed to shed light on their daily lives living with obstetric fistula and how they cope.

**Methods:**

Using a qualitative study design, 19 women living with obstetric fistula were interviewed. The study took place in the fistula ward located in Khartoum hospital and the fistula re-integration center in Khartoum, Sudan. Thematic analysis approach was employed. Stigma and coping theories guided the data collection, analysis, and discussion of the findings.

**Findings:**

Women in our study suffered a challenging physical life due to leakage of urine. In addition, they encountered all forms of stigmatization. Women used both emotion-focused and problem-focused coping techniques to mitigate the consequences of obstetric fistula.

**Conclusion:**

The study findings underscore the importance of obstetric fistula prevention programs and the urgency of repair surgeries to alleviate women’s suffering. Community sensitization, rehabilitation and re-integration of women back to their communities are also important strategies on their journey to wholeness.

## Plain English summary

Pregnancy and childbirth are joyful events in women’s lives. However, this might not be the case for some of them. Women who suffer difficult and long childbirth can end up leaking urine and/or feces. More than two million women are currently living with this condition in the developing world. This devastating condition adversely affects the quality of their lives in terms of their physical, psychosocial and economic wellbeing. There is little research about the experiences of women living with leakage of urine and/or feces in Sudan. Therefore, this article provides a better understanding of how urine leakage affects women’s lives and how they cope with the challenges caused by it. The study took place in the biggest repair clinic in Khartoum, Sudan. Our findings show that women suffered a challenging life due to leakage of urine. They had to live with the continuous wetness, continuous padding and excessive cleaning of their groin area that led to bruises, infections and burning sensations. Getting treated from this condition was difficult for the women especially those who did not know about the existence of the treatment. In addition, they encountered all forms of stigma. Women coped with this condition using techniques that focus on trying to modify both their emotions and their leaking. The study indicates the urgent need for a quick repair, psychosocial support and rehabilitation services to alleviate the suffering of these women and facilitate their integration back to their communities.

## Background

Pregnancy and childbirth should be a joyful experience for women and families. However, many women die or suffer lifelong morbidities due to pregnancy-related causes in low-income countries [1]. Obstetric Fistula (OF) is among the most devastating maternal morbidities. For women who live with OF, the damage is not only physical but also psychosocial, mental and financial.

A fistula is an abnormal opening between the birth canal and bladder and/or rectum that leads to involuntary leakage of urine and/or feces [2–5]. About 90% of cases in resource-poor countries result from an obstetric origin [6, 7]. Most of the OFs happen from obstructed labor due to prolonged lack of blood supply to the walls of the birth canal that develops as a result of fetal head compression of tissues against the pelvic wall [2, 3]. The lack of blood supply kills cells forming a dead tissue that falls away leaving pathological holes that connect the birth canal to the rectum and/or the bladder through which urine and/or feces leak uncontrollably [2, 3]. Severe birth injuries including OF can be prevented through timely access to quality emergency reproductive health services [8].

It is estimated that there are 50,000 to 100,000 new cases of obstetric fistula each year; two to three million women are living with OF mainly in Africa and Asia [2, 9, 10]. Some of the countries with a high burden of OF include Sudan, Ethiopia, Ghana, and Nigeria in Sub-Saharan Africa and Bangladesh from Southern Asia [9]. Obstetric Fistula typically affects marginalized and poor women in rural areas [8].

Obstetric fistula is a multi-dimensional problem that affects women’s lives including their physical, psychological, social and economic wellbeing [2–4]. Women living with fistula suffer from involuntary leakage of urine and/or feces that can result in having sores and ulcers from the irritation of the skin by urine or pads to the perineum and thighs [2]. They also suffer from recurrent infections, may experience total urethral loss, foot drop [9, 11], vaginal narrowing and tears that cause painful intercourse and altered sexual life [5]. These challenges may persist even after fistula repair [11, 12]. Moreover, the fetus is born dead or dies within weeks of delivery in 83% of OF cases which leaves the woman suffering from the aforementioned consequences as well as the grief caused by the loss of her child with or without the ability to conceive again [8, 13]. Thus, affected women may end up losing their identity as women because they cannot perform what is expected of them by their husbands and society [13].

The repair of OF is not merely the closure of the hole alone; the woman must be able to control urination [14]. It also includes women being reintegrated into their communities through rehabilitation and reintegration services [14]. Unfortunately, women who have OF in resource-poor countries most likely have a complicated OF [15]. Meaning that either the lesion is more than six centimeters, multiple lesions, or that the lesion is near to the neck of the bladder which affects continence and the degree of scarring [15]. According to research, operation by a skilled surgeon and careful post-operative care determine the success of the repair surgery with a success rate of 85–90% [8, 16]. However, some of the women will still suffer from some leakage depending on the degree of damage and scarring that happens after repair operation [8]. Despite the huge impact of leaking on women living with OF, treatment is not prioritized in some of the countries where OF is most prevalent [14].

The prevalence of OF in Sudan is unknown due to the lack of prevalence studies. However, it is estimated to be high with 5000 new OF cases occurring annually [17, 18]. A study conducted in Sudan showed that poverty, illiteracy of women in Sudan and early marriage were risk factors where approximately 60% of the study sample were married before the age of 18 and 75% of the women were illiterate [17]. In the same study, nearly half of the women with OF who participated in the study did not go to antenatal care and 42% delivered at home [17]. About 60% of women delivered at a hospital by forceps delivery or by emergency cesarean section [17].

There are few published studies that have examined OF in Sudan; even fewer describing the experiences of women living with fistula. Therefore, this study aimed to examine the impact of OF on the lives of Sudanese women living with it and to identify and understand the coping strategies used to mitigate OF’s effects.

### Theoretical frameworks

In this study, we used the Stigma theory by Goffman and the transactional model of stress and coping by Lazarus and Folkman to analyze and discuss the findings. Goffman describes stigma as “an attribute that is deeply discrediting” [2]. Each society defines what is normal and discredited, and gives a social identity for each member [3]. Failure to adhere to the normative criteria of this social identity may cause the person to be labeled as having a spoiled identity, labeled as an ‘other’ and consequently devalued [3]. Certain conditions such as leakage of urine and/or feces are considered stigmatizing due to the presence of the physical features or deviated behavior according to the standards of the specific community [2, 19, 20]. The process of devaluation i.e. stigmatization is dynamic and it can continuously change and be reshaped by coping strategies adopted by the labeled person [21]. Social integrity can be restored, i.e. the process can reverse itself if the person does not have the discrediting condition. Conversely, stigmatization can become worse if symptoms persist or worsen [20]. Stigma can be self, anticipated or enacted. Self-stigma is the internalized feelings of shame by the labelled woman, anticipated stigma is the fear of discrimination due to a stigmatizing condition and enacted stigma is explicit maltreatment or negative reactions towards the women, in this case, due to merely having the stigmatizing condition of OF [20, 22].

Coping refers to the cognitive or behavioral tactics used by the person to tolerate or mitigate the effects of a stressor, in this case OF and the associated stigmatization [23]. According to Lazarus and Folkman, when a person faces a stressful situation, he/she measures the magnitude of the stressor if it is manageable, endurable, or irrelevant [24]. People also evaluate their abilities to stand against a particular stressful condition [24]. The transactional model of stress and coping conceptualizes two types of coping approaches; problem-focused and emotion-focused coping strategies [25]. According to the intensity of the stressor and the perceived self-abilities to handle the situation; people can use methods to alter the source of stress, i.e. problem-focused coping if the stressor is controllable or they can regulate the emotion elicited by the stressor if it is relatively unchangeable or intolerable [26]. One of the coping responses can predominate according to the intensity of stressor along with losses or harms that can be caused by it [23].

### Methodology

#### Study design and setting

We conducted an exploratory phenomenological qualitative study from a social constructionist perspective [27]. This approach was deemed appropriate because we sought to understand how women relate to and interpret their experiences of how OF impacted their lives since meanings and experiences are socially constructed in relation to their social context. Our study took place in Dr. Abbu Fistula Center (AFC) and the fistula re-integration center (the fistula home). The study sites were selected because they receive fistula cases from all States in Sudan. The fistula center relies on two doctors and a retired surgeon, twelve nurses and one social worker. When the study took place, the clinic had two floors, the first floor contained two postoperative rooms, and an operating theatre with a small room attached where the examination of patients takes place. The upper floor had a waiting room for women who were awaiting their operations. Four to six women are operated on a weekly basis in AFC. The reintegration center is locally called ‘Fistula home’. This center offers pre and post repair, physical and reintegration programs with vocational training and literacy classes. Some women use it as a residence while waiting for treatment.

#### Study participants

The women who lived with OF were central in describing their experiences. Therefore, purposive sampling was used to recruit participants. Purposive sampling means that a researcher selects specific group from a population to join the study according to given criteria to ensure that participants will give insights that are related the most to the phenomenon being studied [28]. Twenty-one women were initially identified by the social worker as eligible to join the study. Nineteen women diagnosed with OF were with two women declining the invitation to participate. To participate, the women had to be: clinically diagnosed with OF, attending AFC or the reintegration center but still not operated on i.e. still leaking, and willingly consent to participate in the study. The study participants were interviewed by the first author. In order to explore the different experiences of women, maximum variation sampling in study participants was ensured. Women from different age groups, literacy levels, ethnic backgrounds, marital status and severity and duration of OF were selected.

#### Sample recruitment

The first author approached the director of AFC and fistula home to conduct the study. The director of the center agreed to the facility participating in the study, and he assigned the social worker to introduce the first author to eligible participants. The first author spent some time at the AFC to familiarize herself with the setting and to gain the trust of the women. Those who were identified as eligible for the study were approached first by the social worker to seek their initial acceptance and then consent was sought by the first author. The first author described in brief the objectives, benefits and possible risks, respondents’ rights and confidentiality measures and asked for their consent to participate voluntarily in the study. Seventeen women were purposively selected from the AFC while two participants were recruited from the fistula reintegration center.

#### Data collection

Face to face in-depth interviews were used. The interviews lasted between 25 and 60 min. Participants chose the interview venue. All of them preferred to have their interviews in situ; so the first author used the office of the social worker to conduct the interviews. The interview guide contained areas to be covered that are central to the research objectives and it was used to guide the discussion rather than to limit it. The areas discussed during interviews included the consequences of OF and coping mechanisms. The interviewer used open-ended questions. Probing questions were used to encourage respondents to talk more about the topic or to gain clearer responses. The first author concurrently made notes about the non-verbal signs made by the interviewees. Most of the interviews were recorded with the consent of the participants. Where participants declined to be recorded, short notes were taken by the first author. In addition, field notes were taken by the first author to remind her about thoughts encountered or to highlight some information during the interviews. Interviews were conducted in Arabic since it is the mother tongue for the majority of the Sudanese people, including the first author, and the official language of the country.

#### Data management and analysis

Audio recordings were transcribed verbatim in Arabic along with notes taken by the first author during fieldwork. That gave her the chance to return to study participants for member checking. The first author then translated transcripts into English. The quality of translation was checked by a colleague that is fluent in both languages by doing backward and forward translation. The analysis process started during the data collection phase while still in the field by noticing patterns of potential interest. Through an iterative process, the transcripts were read carefully to form a general impression of what participants said about their experiences of living with OF. The transcripts were then re-read repeatedly to gain familiarity with the text and to understand the context in which participants lived and coped with OF. We employed a theoretical thematic analysis. We coded manually all segments of the text that capture an idea related to our study questions using memo writing and highlighting data segments. Nonetheless, three transcripts were randomly selected and given to four Masters students at the University of Oslo to code, they provided similar codes. These emergent codes were compared across transcripts and categorized in an excel sheet. The analysis was both deductive and inductive in nature. Deductively, we searched for themes derived from the stigma theory by Goffman and the transactional model of stress and coping by Lazarus and Folkman described in the theoretical framework section above. Themes included consequences of OF, the dimensions of stigmatization, and the coping strategies that women used. Although deductive analysis informed the main themes, inductive analysis informed naming of some emergent sub-themes for example the use of smoke bath as a coping strategy. These sub-themes had not been mentioned before in previous studies and the theoretical frameworks. The stigma theory and transactional model of stress and coping by Lazarus and Folkman helped determine the categorization of emergent codes and sub-themes into the main themes. The themes and the sub-themes were interpreted further and compared to the objectives of the study to generate the final conclusion. The guidelines adopted from both the Standards for Reporting Qualitative Research and the Consolidated Criteria for Reporting Qualitative Research were followed in writing the study report [29, 30].

#### Reflexivity

The first author is somewhat familiar with the setting from where women came since she grew up in the same country and speaks the same language. That made her an insider to some extent. Women may have found it easier and more comfortable to share their deepest thoughts with an insider. At the same time, being an insider might have influenced the way she perceived information that women shared to take some issues as normal or for granted. The first author spent quality time before she started to conduct interviews with women in order for them to become familiar with her and her presence there. The first author is a medical doctor and, in Sudan, the relationship between doctor and patient is authoritative and it potentially would have affected the quality of the data collected. To address that challenge, the participants were reminded continuously that participation is strictly voluntary. The study participants found it easy to communicate with the first author because she identified herself as a researcher and a Masters student. The first author never told the participants that she is a doctor and never wore a white coat.

#### Trustworthiness and rigor

To ensure the trustworthiness of the study findings, data collection continued until saturation level was reached i.e. there were no new concepts coming out from data. Reaching the level of data saturation is important to enhance the trustworthiness of research and it is concerned with the depth of data obtained rather than the number of participants [31]. Audio recordings, member checking and feedback loops from the participants on transcripts ensured that quotations used accurately reflected the voices of the women thereby improving the credibility of the findings and interpretations.

Because the experiences of Sudanese women living with OF provided a rich text, in addition, the study participants, settings and the methodology used were described in as great details as possible, transferability of study methods to other similar contexts may be easily determined.

## Findings

### Characteristics of the study participants

A total of nineteen women living with OF participated in this study. Their ages ranged from 16 years old to 58 years old with an average age of 31 years old. Regarding their marital status, eight of the participants were married, six were divorced, three were abandoned by their husbands i.e. left without an official divorce and two were widows. The majority of them were illiterate (*n* = 11) or left school very early (*n* = 4). Three of the participants had OF for less than a year whereas the rest had it for a duration ranging from two to 18 years. The demographic characteristics of women are summarized in Table 1.

**Table 1:** Characteristics of women

Pseudonym	Age (years)	Educational Level	Marital Status	Years (y) /Months (m) with OF	Number of Surgeries
Sakeena	24	Illiterate	Divorced	4 y	2
Aisha	16	Primary school	Married	5 m	0
Haleema	32	Illiterate	Divorced	4 y	0
Fatima	30	Illiterate	Abandoned	7 y	0
Marwa	20	Illiterate	Divorced	10 m	0
Altaya	17	Primary school	Married	8 m	0
Sara	20	Illiterate	abandoned	1 y	0
Mona	18	Illiterate	Married	1 y	1
Samia	58	Teachers Institute	Widow	18 y	1
Sit Albanat	45	Illiterate	Married	15 y	2
Maha	18	Illiterate	Abandoned	1.5 y	2
Zeinat	27	University – 1st year	Married	6 y	6
Asma	47	Primary school	Married	7 y	2
Sitana	50	Illiterate	Widow	15 y	1
Amira	27	Illiterate	Divorced	12 y	3
Khadeeja	33	Midwifery school	Married	5 y	1
Maysa	23	Primary school	Divorced	7 y	1
Ensaf	33	Illiterate	Divorced	17 y	11
Tahani	30	University	Married	2 y	1

Our study participants had to live with continuous wetness that resulted from OF, continuous padding and excessive cleaning of their groin area that lead to bruises, infections, and burning sensations. The findings are categorized below according to two major themes: women’s experiences of stigmatization which includes their experiences with anticipated, self and enacted stigma; and the coping strategies that they used to mitigate the effect of OF which includes emotion and problem-based coping strategies.

### Women experiences with stigmatization

#### Experiences of women with anticipated stigma

Our study participants lived with OF for periods that reached up to 18 years. Getting treated from OF was difficult for the women especially those who did not know about the existence of the treatment. Several women stated:

“*I stayed for seven years and nobody told me that my condition can be treated. Nobody told me what to do I figured it out myself*”. [Fatima, 30 years old, seven years living with OF]

*“I was just sitting [staying as she is] because I didn’t have hope for treatment”* [Maysa, 23 years old, seven years living with OF]

Many women in our study were afraid that others might discover that they are leaking urine because of their smell and humiliate, isolate, think or talk badly about them. Therefore, women suffering from OF were obliged to isolate themselves and forsake social gatherings because they expected that someone would talk about the smell as expressed in the quotes below:

*“I used to go to weddings, visit the neighbors but now I just stay at home, I don't go outside at all. All the people go out and leave me alone. I don’t want to go out because I am shy because of my condition [OF]. I say to myself that I have fistula where would I dare to go, I am 24 hours a day at the bathroom or changing my clothes”* [Mona, 18 years old, one year living with OF]

In some cases, the anticipated stigma happened after witnessing or having an incident of enacted stigma.

“*I didn't hear but when I walk by someone I say to myself what if that person can smell an odor on me, I have to think like that, I just tell you the truth … I heard also people talking about a woman who has had a fistula. They said ‘this woman smells bad. Why doesn't she go to treat herself?' They cannot sit with her and if they do you find them agitated as if they cannot stand her anymore … I say to myself that if I walk by them they will say the same thing about me.”* [Haleema, 32 years old, four years living with OF].

#### Experiences of women with self-stigma

Many of the women living with OF suffered from low self-esteem and worthlessness. They internalized negative self-perceptions such as low self-esteem and the sense of being worthless. A participant conveyed her feelings in the following quotation:

*“I feel myself incomplete, and I am inferior to people. I see all people better than me because I have this illness, nobody had it before I got it”* [Mona, 18 years old, one year living with OF].

Other participants stated:

“*I am never like I was in the past, I see myself as if my status is lower now. When I look at myself now I see myself different. When I join people in conversation, nobody listens to what I say like before, they see me like a weightless person*”. [Khadeeja, 33 years old, five years living with OF]

*“I feel myself deficient and not like other people because I see all of them work in the farms and I sit here doing nothing I feel myself useless and ill.”* [Maha, 18 years old, 1.5 years living with OF].

#### Women’s experiences with enacted stigma

##### Divorce and abandonment due to OF

Almost half of the study participants were divorced or abandoned by their husbands as a result of getting obstructed fistula. Two participants divulged:

“*I told him [about the fistula] after we got back to the village, he left us at the hospital. He didn’t say anything to me, he just came after a week and said that I divorced you. What can I say, he just divorced me because of what has happened to me.*” [Haleema, 32 years old, four years living with OF]

*“He told me that I will not be well again, after I went home he sent someone to say that he left me. He left me because I became ill”* [Amira, 27 years old, seven years living with OF]

##### Verbal insults

The majority of the women living with OF were victims of verbal insults from their relatives due to their condition. They were mocked by their husbands and family for being smelly and dirty all the time and being unable to do house chores. Some mentioned that people would advise the husband to leave them because of their condition. Women used terms like different, defective, deficient, incomplete to describe how others would think about them because of their condition. A participant shared the following:

“*Even if a small discussion happened they name you by it [urine] and say to you “the one with urine” or the stinky one. No respect for me and they don’t consider me a human. You are leaking urine then you are not valued in the community*”. [Zeinat, 27 years old, six years living with OF]

Another said:

“*Since I had this fistula I have had problems with my husband and his family. They told me that I am useless. They say that I have become useless because I can’t have normal labor, sit with other people or cook food. They say that I rush to the bathroom every now and then while cooking* … *When I cook my in-laws tell me that I am ill and maybe I didn’t wash my hands or didn’t have a bath so they cannot eat what I have cooked. They say that to me all the time. I just gave up”*. [Maysa, 23 years old, seven years living with OF]

### Coping strategies

#### Emotion-based coping

##### Acceptance through fate

Getting OF and losing the baby were some of the adverse events that women had to deal with. The majority of women talked about OF being their fate or that it was destined from Allah “God” and they had to accept it no matter how bad it was. This belief is expressed in the quotes below:

“*Rabbana [Our God] gave it to me, what can I say?*” [Sakeena, 24 years old, four years living with OF]

“*I say that this is what Allah wants for me and there is no way out even if I get angry or I cry; there is no way other than patience … If something is planned by Allah, It will never leave you, we just thank Allah for it.”* [Haleema, 32 years old, four years living with OF]

#### Fistula Ward/home as a refuge

Some of the participants preferred to stay at the hospital or the fistula home because they felt more comfortable there; they made an alternative home and family. Women also found the solidarity in the company of each other and felt better that they were not alone.

“*I stay here because all the people here are ill like me, we are all the same here. There [Her own home] I don’t feel equal to others … Here is better because I have these women with me, I don’t feel that I am ill or sad … Sometimes I say that I shouldn’t go anywhere, I become better after I came here.*” [Amira, 27 years old, 12 years living with OF]

#### Silence and self-isolation

As reported earlier, many participating women encountered unpleasant and sometimes very extreme reactions from others. Some of them chose to remain silent in order to cope with those events as described by the following quotation:

“*What can I do! I will just be silent. If one treats me well I deal with him if not I just shut up.*” [Sakeena, 24 years old, four years living with OF]

Isolation could be both a challenge that women have to live with when people stay away from them and a way of coping when they self-isolate. In a related manner, some chose distance; one participant elaborated:

“*I just distant myself because I am afraid that someone might hurt me or embarrass me or say bad things to me.*” [Maysa, 23 years old, seven years living with OF].

#### Emotional support

Support from a husband, close family members, and peers who went through the same problem helped women to survive OF. However, the majority of women included in this study did not receive support from their husbands. One participant had tremendous support, especially emotionally, from her late husband which helped her to cope. She described the support in the following quote:

“*My husband was supportive to me to his last moment of life. He didn't let me down not even a second. He never looked at me as a damaged woman until he died. He was my main support system and he never abandoned me. If there was divorce or a hate problem, fistula would have affected me but I never encountered that”* [Samia, 58 years old, 18 years living with OF]

### Problem-based coping

#### Concealment of their condition

Frequent changing of clothes, washing and putting pads made from pieces of old clothes were done by some women to conceal their condition and mitigate the social consequences and personal discomfort associated with OF. In addition, they used scents to cover the smell as long as they could. Study participants divulged:

“*… I bath a lot and use perfume and wash with soap a lot. If someone visits me at home he/she would never be able to tell that I have fistula unless I say so, because he won’t find a smell.*” [Asma, 47 years old, seven years living with OF]

“*If I am wet I go wash that piece of cloth and put it again. I use a light one so it is easily washed and gets dry faster … When you can organize yourself, and hide something like this, it is good.*” [Sitana, 50 years old, 15 years living with OF]

Few women were able to conceal their condition which protected them from verbal insults and discrimination. One participant was able to hide her condition even from her kids and siblings for years and her husband was the only person who knew about her illness. This had protected her from being discovered. She stated:

“*They [everyone around her] don’t know nor they smell something so that they offend me … If I said that I have fistula even if I don’t smell, people will pay attention, but I didn’t say*” . . . “*When I go to school, I put my dirty pad in multiple bags, I wait till 12 o'clock to go to the bathroom because people are at classes at that time. So I go to the bathroom and change, I wrap the dirty pad in a number of plastic bags so if someone saw it will think that it is a private matter. This was my biggest worry that other teachers open my bag to take a pen and see the dirty pad that's why I wrap it that way … I wash my clothes in a distant place in my house away of people sights, I have a specific place to dry my clothes because of my children and guests*” [Samia, 58 years old, 18 years living with OF]

#### Drinking less water

A few working women mentioned that they drank less water at work to minimize the amount of urine dispensed. However, drinking less water caused complications for some of them such as infections and kidney stones. Participants said:

“*Drinking water annoys me because of wetness and this caused me urinary infections. When I came here this time, they requested an image because I might have a stone because I don't drink water while working*”. [Khadeeja, 33 years old, five years living with OF]

“*When I have breakfast at school I don’t drink water much, they give me water and I take a sip and I tell them I had enough.*” [Samia, 58 years, 18 years living with fistula]

#### Smoke bath

Some women used fumigation by sitting on wood smoke that helped them cover the smell and reduce the amount of urine leakage. One participant elaborated:

“*I just use dokhan [smoke bath] and diaper, then nothing would be on me. I use dokhan [smoke bath] morning and evening then the smell will be gone and no leakage. It makes the urine amount less even if it leaked the smell will be covered. When I go to the bathroom the smell will be like the smell of smoke*”. [Khadeeja, 33 years old, five years living with OF]

This participant claimed that smoking twice every day made her life as normal as it could be, especially since she was able to get the wood she used easily from where she lived.

## Discussion

In the current study the aim was to glean insights into the lives of the participating Sudanese women living with OF, explore their experiences and how they coped day in and day out. The study revealed similar lives yet varying forms of stigma experienced among the participants and various coping strategies to mitigate the physical and psychological sequelae of OF. The findings indicate that as a result of urine leakage and its smell, the majority of our study participants lived stigmatized lives because of lack of control of a bodily function “urine”. Women had to live with all dimensions of stigma due to leakage and the smell of urine. Due to the common stereotypes related to loss of control of bodily functions in addition to enacted stigma, women internalized feelings of worthlessness and shame and they feared discrimination i.e. anticipated stigma. Women coped with the effects of OF with social isolation and silence. In addition, they tried to conceal their condition by hiding the urine smell by frequent bathing, using scents and smoke baths.

The concept of embodiment refers to the skillful mastery of bodily functions to situate the body in social contexts for example by learning to follow a socially acceptable way to eliminate bodily wastes such as urine, feces, flatus, etc. [32]. This is because cultures define urine and feces as polluting substances that need to be disposed in private, and in a proper timing to correspond with social norms [21]. Learning the skills of controlling these bodily functions are acquired early in life [33] and shortcomings in doing so jeopardize and breach the boundaries of the embodied self as described by Goffman [32]. It affects self-presentation and the social competency of being viewed as self-sufficient which leads to embarrassment up to the loss of self-esteem and other psychosocial effects [32]. Deviation from the social norms is a cause of stigma, for example through the inability to control bodily functions in the case of OF. Therefore, it can be argued that women in our study have deviated from what is perceived to be “normal” in Sudanese society as well as in other African countries. Therefore, they were labeled as different with a spoiled identity [2]. As such, the smell of urine and leaking itself was severely discrediting and stigmatizing for women as it was a source of shame, low self-esteem, anxiety, embarrassment and a cause of humiliation [3]. By leaking urine and feces, women were seen as dirty, smelly, and unable to physically do house chores.

People are exposed, as part of socialization, to common stereotypes related to the inability to control bodily functions such bedwetting early in life [33]. So if later they happen to get into a similar situation, how others think about them becomes relevant which provides a ground to internalize those devaluing views [19]. Women in our study internalized what their communities prejudiced about them regarding worthlessness and shame and they saw themselves as dirty, defective and incomplete. In addition, they anticipated discrimination in light of their previous knowledge of those stereotypes. On the other hand, verbal insults and discrimination were utilized by others to discipline and remind women of their lower status by calling them stinky and other devaluing descriptions [34]. It was observed in this study that the fear of discrimination i.e. anticipated stigma is connected to having previous experiences with enacted stigma and witnessing other affected women suffer rejection or discriminatory behaviors due to their condition [21]. Therefore, some women tried to conceal their situation by making vigorous efforts to stay clean and hide the smell of urine to protect themselves from potential devaluation. Although few women were able to conceal their smell, the majority of the women could not conceal their condition. These findings contradict findings from a study in Uganda which found that many who had OF were able to conceal their leakage and smell and that was effective in protecting themselves from enacted stigma [35].

According to our findings, many of the affected women waited for long periods of time before seeking treatment. Similar findings have been observed elsewhere [2]. Women in our study reported that the lack of knowledge about the availability of repair surgeries as a reason for treatment delay, an observation reported in Ghana [3]. A systematic review indicated that seeking treatment for mental illnesses was associated with treatment stigma and felt stigma [36]. Women living with OF can be faced with the same experience while seeking repair surgery.

Leakage and the urine smell that constantly haunted the women living with OF constituted a stressful condition with which they needed to cope. As asserted by Lazarus and Folkman, women in our study used both emotion- and problem-based coping. Lazarus and Folkman suggested that the choice of the coping mechanism is dependent on the magnitude of the stressing factor and the perceived internal strength to stand against it. Coping strategies can be adaptive or maladaptive in relevance to the outcomes related to physical and psychosocial wellbeing [25]. For instance, women in our study and other studies used silence and self-isolation to cope with mocking and negative encounters and to protect themselves from stigma [2, 35]. However, it can be argued that these strategies might worsen their psychosocial status, increase stress and lower their self-esteem and self-confidence instead of healing [25]. In addition, they led to loss of contact with friends and worsened detachment from their social networks. A previous study that discussed the effect of environment on coping has indicated that individuals are less likely to cope with a stressful condition with withdrawal and isolation if they are surrounded by a supportive environment [24]. In addition, one cannot really know if self-isolation is consistently used by women as a coping strategy or rather as a result of the failure of the coping processes which led to dissolution. Some of the activities that women use to cope with their condition such as excessive cleaning and continuous padding may aggravate their physical impairment due to the development of sores and burning sensation in their private parts [2].

Similarly, the women in our study adopted problem-focused coping mechanisms. Activities such as frequent bathing, using scents and smoke were used by women to remove the urine smell where they were using pads to maintain their dryness. Through eliminating the smell, the participants potentially avoided stigmatization. These mechanisms, apart from the smoke bath, are similar to what was reported among women in Nigeria, Tanzania, and Uganda [2, 37–39]. Seeking social support as a way of coping is rather dependent on the context. In our study, women felt isolated in their homes, yet, they found comfort in the company of other women living with OF. Therefore, they found it better to stay at the hospital or fistula home. A study conducted in Kenya suggested that the company of other women with OF might have protected women from isolation and as such, improving their mental health status [7]. Moreover, the company of women having the same condition at the fistula clinic in Addis Ababa helped women orientate to their realities and gave them strength and hopes for recovery [23].

Like the majority of the Muslim community, women in our study expressed a strong reliance on God’s will, including coping with the consequences of OF which was consistent with what was observed in Tanzania [40]. They prayed for treatment and strength to live the difficult life that they have. Adverse events such as losing a child or suffering from a serious disease were seen as a test from God, as well as, people being rewarded if they showed patience [41]. The use of religious coping was common among women in our study which is consistent with what was observed in Tanzania and Uganda [35, 40]. In contrast to what was described in the Tanzanian context, religious leaders ‘Imams’ did not have an active role in women’s journey to look for treatment [40]. Lack of use of religious leaders by the health system is a missed opportunity to reach women, their families, and their communities by health education and psychological support that can enhance their opportunities to get treatment.

### Study strengths and limitations

The study gives a rich description of the lived experiences of women living with OF. The rich descriptions of the study settings, the Sudanese women living with OF and methods enhance transferability of study findings to similar contexts. This study aimed at a rich description of the challenges that women faced due to OF and the strategies they used to mitigate those consequences. Despite the appropriateness of the study design, triangulation of other sources of information from husbands, family members, and health workers would have strengthened the findings by providing a more comprehensive picture of the context of the other actors who play a role in either perpetuating or mitigating the consequences of OF. In future research, the views of partners, family members, and community members would be insightful. The study was a facility-based study which might have caused selection bias where our study sample were women who managed to reach the treatment facility. Lack of women recruited from the community may unintentionally overlook women who are less supported by their relatives and are likely to suffer from more severe consequences of OF.

## Recommendations for further research

There are areas for future research that would help decrease the ambiguity of the situation of OF in Sudan. One of them is a country-wide prevalence study of OF and the associated risk factors. This would be used in advocacy and to enlighten policy making and priority setting for budget allocation. This study focused on the experiences of women living with OF. There is no doubt that men also encounter challenges when they have wives with OF so it would be helpful to shed light on the experiences of these men. Experiences of women with OF in the community who do not seek or fail in their attempts to seek care was beyond the scope of this study, but such community-based studies are warranted if OF is to be addressed throughout Sudan. Those women might have more severe consequences of OF.

## Conclusion

Having OF is not merely having a hole that leaks urine and/or feces uncontrollably, but instead disruptive interpersonally inside the home and in the community. Based on challenges narrated by the women in this study, the urgency of repair surgeries should be taken more seriously and made available widely in order to alleviate unnecessary and preventable suffering. The issue of OF and women’s wellbeing must also be prioritized in the national agenda and more resources to be allocated to strengthen the quality and coverage of services for treatment and re-integration of women affected by OF. Also, programs that promote the involvement of the community in the issue of OF are beneficial for the detection of new cases, mitigation of stigma and the improvement of support (moral, spiritual and financial support).

## Data Availability

The data that support the findings of this study are available from the corresponding author upon reasonable request.

## Abbreviations

AFC: Dr. Abbu Fistula Centre
MoH: Ministry of Health
OF: Obstetric fistula

## References

[1] Victora CG, Requejo JH, Barros AJD, Berman P, Bhutta Z, Boerma T (2016). Countdown to 2015: a decade of tracking progress for maternal, newborn, and child survival. Lancet.

[2] Barageine JK, Beyeza-Kashesya J, Byamugisha JK, Tumwesigye NM, Almroth L, Faxelid E (2015). “I am alone and isolated”: a qualitative study of experiences of women living with genital fistula in Uganda. BMC Womens Health.

[3] Mwini-Nyaledzigbor PP, Agana AA, Pilkington FB (2013). Lived experiences of Ghanaian women with obstetric fistula. Health Care Women Int.

[4] Yeakey MP, Chipeta E, Taulo F, Tsui AO (2009). The lived experience of Malawian women with obstetric fistula. Cult Health Sex.

[5] Hamed S, Ahlberg B-M, Trenholm J (2017). Powerlessness, normalization, and resistance: a Foucauldian discourse analysis of Women’s narratives on obstetric fistula in eastern Sudan. Qual Health Res.

[6] Cook RJ, Dickens BM, Syed S (2004). Obstetric fistula: the challenge to human rights. Int J Gynecol Obstet.

[7] Weston K, Mutiso S, Mwangi JW, Qureshi Z, Beard J, Venkat P (2011). Depression among women with obstetric fistula in Kenya. Int J Gynecol Obstet.

[8] Mselle LT, Moland KM, Evjen-Olsen B, Mvungi A, Kohi TW (2011). " I am nothing": experiences of loss among women suffering from severe birth injuries in Tanzania. BMC Womens Health.

[9] Miller S, Lester F, Webster M, Cowan B (2005). Obstetric fistula: a preventable tragedy. J Midwifery Womens Health.

[10] Siddle K, Mwambingu S, Malinga T, Fiander A (2013). Psychosocial impact of obstetric fistula in women presenting for surgical care in Tanzania. Int Urogynecol J.

[11] Drew LB, Wilkinson JP, Nundwe W, Moyo M, Mataya R, Mwale M (2016). Long-term outcomes for women after obstetric fistula repair in Lilongwe, Malawi: a qualitative study. BMC Pregnancy Childbirth.

[12] Khisa AM, Nyamongo IK (2012). Still living with fistula: an exploratory study of the experience of women with obstetric fistula following corrective surgery in west Pokot, Kenya. Reprod Health Matters.

[13] Ahmed S, Anastasi E, Laski L (2016). Double burden of tragedy: stillbirth and obstetric fistula. Lancet Glob Health.

[14] De Bernis L. Obstetric fistula: guiding principles for clinical management and programme development, a new WHO guideline. Int J Gynecol Obstet. 2007;99:S117–21. 10.1016/j.ijgo.2007.06.032 17880979

[15] Genadry R.R., Creanga A.A., Roenneburg M.L., Wheeless C.R. (2007). Complex obstetric fistulas. International Journal of Gynecology & Obstetrics.

[16] Shittu O, Ojengbede O, Wara L (2007). A review of postoperative care for obstetric fistulas in Nigeria. Int J Gynecol Obstet.

[17] Mohamed E, Boctor M, Seedahmed H, Abdelgadir M, Abdalla S (2009). Contributing factors of vesico-vaginal fistula (VVF) among fistula patients in Dr. Abbos National Fistula & Urogynecology Centre-Khartoum 2008. Sudanese J Public Health.

[18] Vangeenderhuysen C, Prual A, el Joud DO (2001). Obstetric fistulae: incidence estimates for sub-Saharan Africa. Int J Gynecol Obstet.

[19] Link BG, Phelan JC (2013). Labeling and stigma. Handbook of the sociology of mental health: Springer.

[20] Goffman E (2009). Stigma: notes on the management of spoiled identity: Simon and Schuster.

[21] Changole J, Thorsen VC, Kafulafula U (2017). “I am a person but I am not a person”: experiences of women living with obstetric fistula in the central region of Malawi. BMC Pregnancy Childbirth.

[22] Heijnders M (2004). The dynamics of stigma in leprosy. Int J Lepr Other Mycobact Dis.

[23] Gebresilase YT (2014). A qualitative study of the experience of obstetric fistula survivors in Addis Ababa, Ethiopia. Int J Womens Health.

[24] Parkes KR (1986). Coping in stressful episodes: the role of individual differences, environmental factors, and situational characteristics. J Pers Soc Psychol.

[25] Lazarus RS (1993). Coping theory and research: past, present, and future. Fifty years of the research and theory of RS Lazarus: An analysis of historical and perennial issues.

[26] Quine L, Pahl J (1991). Stress and coping in mothers caring for a child with severe learning difficulties: a test of Lazarus' transactional model of coping. J Community Appl Soc Psychol.

[27] Holstein JA, Miller G (2006). Reconsidering social constructionism: debates in social problems theory: transaction publishers.

[28] Marshall MN (1996). Sampling for qualitative research. Fam Pract.

[29] Tong A, Sainsbury P, Craig J (2007). Consolidated criteria for reporting qualitative research (COREQ): a 32-item checklist for interviews and focus groups. Int J Qual Health Care.

[30] O’Brien BC, Harris IB, Beckman TJ, Reed DA, Cook DA (2014). Standards for reporting qualitative research: a synthesis of recommendations. Acad Med.

[31] Fusch PI, Ness LR (2015). Are we there yet? Data saturation in qualitative research. Qual Rep.

[32] Weinberg MS, Williams CJ (2005). Fecal matters: habitus, embodiments, and deviance. Soc Probl.

[33] Mota DM, Barros AJ (2008). Toilet training: methods, parental expectations and associated dysfunctions. J Pediatr.

[34] Link BG, Phelan J (2014). Stigma power. Soc Sci Med.

[35] Kabayambi J, Barageine JK, Matovu JK, Beyeza J, Ekirapa E, Wanyenze RK (2014). Living with obstetric fistula: perceived causes, Challenges and Coping Strategies among Women Attending the Fistula Clinic at Mulago Hospital, Uganda.

[36] Clement S, Schauman O, Graham T, Maggioni F, Evans-Lacko S, Bezborodovs N (2015). What is the impact of mental health-related stigma on help-seeking? A systematic review of quantitative and qualitative studies. Psychol Med.

[37] Okoye UO, Emma-Echiegu N, Tanyi PL. Living with vesico–vaginal fistula: Experiences of women awaiting repairs in Ebonyi state, Nigeria. Tanzania J Health Res. 2014;16(4). 10.4314/thrb.v16i4.9 26891522

[38] Mehta M, Bangser M, Barber N, Lindsay J, Gujrati M (2007). Sharing the burden: Ugandan women speak about obstetric fistula.

[39] Mselle LT, Kohi TW (2015). Living with constant leaking of urine and odour: thematic analysis of socio-cultural experiences of women affected by obstetric fistula in rural Tanzania. BMC Womens Health.

[40] Watt MH, Wilson SM, Joseph M, Masenga G, MacFarlane JC, Oneko O (2014). Religious coping among women with obstetric fistula in Tanzania. Global Public Health.

[41] Hedayat K (2006). When the spirit leaves: childhood death, grieving, and bereavement in Islam. J Palliat Med.

