# Correction to: “I feel myself incomplete, and I am inferior to people”: experiences of Sudanese women living with obstetric fistula in Khartoum, Sudan

**DOI:** 10.1186/s12978-020-0886-3

**Published:** 2020-02-19

**Authors:** Salma A. E. Ahmed, Viva C. Thorsen

**Affiliations:** Institute of Health and Society, Department of Community Medicine and Global Health, University of Oslo, P.O. Box 1130, Blindern, 0318 Oslo, Norway

**Correction to: Reprod Health**

**https://doi.org/10.1186/s12978-019-0846-y**

Following publication of the original article [1], we have been notified that one of the authors’ names was mentioned twice. Currently the authors are stated as:

Salma A. E. Ahmed, Viva C. Thorsen and Salma A. E. Ahmed*

It should be:

Salma A. E. Ahmed*, Viva C. Thorsen
